# Disorder- and emotional context-specific neurofunctional alterations during inhibitory control in generalized anxiety and major depressive disorder

**DOI:** 10.1016/j.nicl.2021.102661

**Published:** 2021-04-03

**Authors:** Congcong Liu, Jing Dai, Yuanshu Chen, Ziyu Qi, Fei Xin, Qian Zhuang, Xinqi Zhou, Feng Zhou, Lizhu Luo, Yulan Huang, Jinyu Wang, Zhili Zou, Huafu Chen, Keith M. Kendrick, Bo Zhou, Xiaolei Xu, Benjamin Becker

**Affiliations:** a The Clinical Hospital of Chengdu Brain Science Institute, MOE Key Laboratory for NeuroInformation, High-Field Magnetic Resonance Brain Imaging Key Laboratory of Sichuan Province, University of Electronic Science and Technology of China, Chengdu, Sichuan 610054, China; b Chengdu Mental Health Center, Chengdu, Sichuan 610036, China; c Department of Psychosomatic Medicine, Sichuan Academy of Medical Sciences & Sichuan Provincial People’s Hospital, Chengdu, Sichuan 610072, China

**Keywords:** Generalized anxiety disorder, Major depressive disorder, Biomarker, Emotion, Inhibitory control

## Abstract

Major Depressive Disorder (MDD) and Generalized Anxiety Disorder (GAD) are highly debilitating and often co-morbid disorders. The disorders exhibit partly overlapping dysregulations on the behavioral and neurofunctional level. The determination of disorder-specific behavioral and neurofunctional dysregulations may therefore promote neuro-mechanistic and diagnostic specificity. In order to determine disorder-specific alterations in the domain of emotion-cognition interactions the present study examined emotional context-specific inhibitory control in treatment-naïve MDD (n = 37) and GAD (n = 35) patients and healthy controls (n = 35). On the behavioral level MDD but not GAD exhibited impaired inhibitory control irrespective of emotional context. On the neural level, MDD-specific attenuated recruitment of inferior/medial parietal, posterior frontal, and mid-cingulate regions during inhibitory control were found during the negative context. GAD exhibited a stronger engagement of the left dorsolateral prefrontal cortex relative to MDD. Overall the findings from the present study suggest disorder- and emotional context-specific behavioral and neurofunctional inhibitory control dysregulations in major depression and may point to a depression-specific neuropathological and diagnostic marker.

## Highlights

• fMRI affective GO/NOGO tasks differentiates depression (MDD) from anxiety (GAD).
• MDD but not GAD showed impaired inhibitory control on the behavioral level.
• MDD exhibited decreased engagement of posterior frontal/mid-cingulate regions.
• The neural alterations were specific for MDD and inhibition in negative contexts.
• GAD showed intact inhibition and enhanced dlPFC activity relative to MDD.

## 1 Introduction

With global prevalence rates as high as 7% (Baxter et al., 2013, Malhi and Mann, 2018), depression and anxiety disorders have become one of the leading causes of disabilities (Whiteford et al., 2013). Comorbidity between them is generally high, with Major Depressive Disorder (MDD) and Generalized Anxiety Disorder (GAD) exhibiting a particular high co-morbidity (Kessler et al., 2008, Maron and Nutt, 2017, Watson, 2009). On the symptomatic level both disorders are characterized by emotional and cognitive dysregulations, including exaggerated negative affect and impaired executive functions (Moran, 2016, Rock et al., 2014, Snyder, 2013). The disorders moreover share therapeutic responsivity (He et al., 2019, Nutt, 2004), genetic risk factors (Kessler et al., 2008), and neural circuit disruptions (Etkin and Schatzberg, 2011, McTeague et al., 2016, Xu et al., 2020b), suggesting partly overlapping neurobiological pathways. On the other hand, disorder-specific phenotypes such as anhedonia (depression) and physiological hyperarousal (specific to anxiety disorders) exist (Clark and Watson, 1991). In line with the differential profiles on the symptomatic level, initial transdiagnostic neuroimaging studies that directly compared MDD and GAD patients revealed disorder-specific neurofunctional alterations (Etkin and Schatzberg, 2011, Xu et al., 2020b, Xu et al., 2020a), which are of particular importance to promote neuro-mechanistic, diagnostic and therapeutic specificity.

On the symptomatic level, key symptoms of both disorders encompass shared dysregulations in emotional and cognitive domains. Patients with MDD as well as GAD exhibit automatic, persistent and uncontrollable negative thoughts about themselves and their future (Ruscio et al., 2011). Neuropsychological case-control studies that compared either MDD, or GAD patients with healthy controls revealed an increased automatic attentional bias toward negative emotional stimuli (Dalgleish and Watts, 1990, Gotlib et al., 2004, Mogg and Bradley, 2005), which may further exacerbate the negative emotional state (Decker et al., 2008, Roemer et al., 2009). Together with the impaired capability to regulate negative emotions the exaggerated reactivity to negative emotional information and accompanying high arousal may critically impede cognitive processing in the domains of attention, memory and cognitive control.

Response inhibition represents an important core component of the cognitive control system and refers to the suppression of prepotent behavioral responses to meet current contextual and task demands (Aron, 2007). Previous studies revealed a lack of inhibitory control of prepotent stimulus–response contingencies across psychiatric disorders (McTeague et al., 2016), suggesting a putative transdiagnostic deficit. However, emotional context- and disorder-specific dysregulations in the interplay between emotion processing and inhibitory control remain poorly understood.

Initial case-control studies examined the influence of emotional context on inhibitory control in MDD by means of affective go/no-go paradigms and reported emotional context-specific control deficits in depressive patients, such that inhibitory control deficits were predominately observed in the context of emotional stimuli. For instance, relative to healthy controls, MDD patients did not exhibit a general cognitive control deficit but presented a mood congruent bias for emotionally salient stimuli (Erickson et al., 2005, Harfmann et al., 2019). In contrast, research on emotional context-specific inhibitory control deficits in GAD has been scarce and revealed rather inconsistent findings, with a recent case-control study reporting enhanced proactive control of negative valence distractors in GAD patients (Hallion et al., 2019).

Likewise, studies employing neuroimaging methods to delineate the underlying neurofunctional basis of inhibitory control deficits in emotional contexts have mainly focused on MDD. For instance, two previous case-control neuroimaging studies employing affective go/no-go paradigms reported that MDD patients showed emotional-context specific aberrant neural engagement of frontal regions (Elliott et al., 2002), and attenuated neural recruitment of the right dorsolateral prefrontal cortex (DLPFC) and bilateral occipital cortex during inhibitory control trials (no-go targets) that followed a negative, but not a positive, stimulus (Colich et al., 2016). Treatment evaluation studies in MDD furthermore reported that administration of non-invasive stimulation to frontal regions normalized emotion-specific cognitive control deficits in depression (Bermpohl et al., 2006, Boggio et al., 2007). Together, these findings suggest that aberrant emotion-cognition integration in frontal regions may underpin the emotional-context specific cognitive control deficits in MDD. In comparison, research on emotional-context dependent neurofunctional alterations in GAD is scarce. One study reported decreased right DLPFC amplitudes in GAD patients compared to healthy controls during inhibition of negative information in an explicit emotional inhibition paradigm, while the patients exhibited intact processing during an implicit emotional inhibition paradigm (Yu et al., 2015).

Overall, the previous findings suggest that deficient inhibitory control in emotional contexts may represent a dysregulation that can differentiate between MDD and GAD and thus represent a behavioral and neurobiological marker with a promising potential to uncover disorder-specific pathological mechanisms. Against this background the present study employed a transdiagnostic design during which patients with MDD or GAD, and matched control subjects underwent an affective (linguistic) go/no-go paradigm with concomitant fMRI acquisition. fMRI was employed to allow the determination of the neurobiological basis of the pathology-relevant dysregulations. Given that recent meta-analyses reported that transdiagnostic impairments in cognitive control are neurally mediated by aberrant recruitment of the fronto-parietal cognitive control networks, as well as the anterior insula, and the midcingulate/presupplementary motor area (Feng et al., 2018, McTeague et al., 2017), and that a growing number of case-control studies suggest separable and emotional context specific cognitive control alterations in GAD and MDD, we hypothesized that MDD and GAD patients manifest distinct emotional context-specific neural impairments.

Based on previous meta-analyses reporting robust impairments in inhibitory control in MDD (Synder, 2013; Rock et al., 2014) as well as a number of original studies reporting valence-specific neurofunctional alterations during negative emotional contexts in MDD (Elliott et al., 2002, Colich et al., 2016) we hypothesized that (1) the MDD group would exhibit impaired inhibitory control; and that (2) MDD patients would exhibit marked neurofunctional alterations in the negative context, specifically deficient recruitment of the frontal cognitive control network as compared to controls. Given the inconsistent findings with respect to implicit emotion regulation in GAD (Etkin and Schatzberg, 2011, Gotlib et al., 2004, Hallion et al., 2019) we hypothesized that GAD patients would exhibit either subtle or no alterations as compared to the healthy reference group reflecting an MDD-specific deficit in emotional-context specific inhibitory control.

## 2 Method

### 2.1 Participants

To control for confounding effects of treatment and changes related to progressive maladaptations during recurrent episodes of the disorders (Treadway et al., 2015, Yüksel et al., 2018) treatment-naïve patients with generalized anxiety disorder (GAD, n = 35) or major depressive disorder (MDD, n = 37) who received their first diagnosis of GAD or MDD respectively within the previous month as well as matched healthy controls (HC, n = 35) were recruited (Exclusion criteria sees Supplementary materials). Demographic data, current levels of depressive and generalized anxiety symptoms were assessed by means of validated questionnaires (BDI-II, PSWQ, (Beck et al., 1996). Given that childhood trauma may affect inhibitory control (Kim et al., 2018, Liu et al., 2020, Maier et al., 2020) the Childhood Trauma Questionnaire (Bernstein et al., 2003) was administered (Table 1). The study was part of a larger project on common and disorders-specific alterations in MDD and GAD, the present paradigm was preceded by resting state fMRI acquisition (Xu et al., 2020a) and followed by a pain empathy paradigm (Xu et al., 2020b). The study was approved by the local ethics committee, adhered to the Helsinki Declaration and written informed consent was obtained before enrollment.

**Table 1:** Demographics, symptom load, and early life stress. PSWQ = Penn State Worry Questionnaire; BDI-II = Beck depression Inventory II; CTQ = Childhood Trauma Questionnaire; EA = Emotional Abuse; EN = Emotional Neglect; PA = Physical Abuse; PN = Physical Neglect; SA = Sexual Abuse; Given that some participants did not completed all questionnaires (details see also: Demographic data and symptom load) the number of subjects who entered the respective analysis is reported for each measure. ^a^ for non-normal distributed data, median and range are reported. **p < 0.01;*** *p* < 0.001, Bonferroni-corrected.

	GAD (N = 26)	MDD (N = 30)	HC (N = 34)				
Male	N = 14	N = 7	N = 12				
	Mean(*SD*)/Median(Range)	Mean(*SD*)/Median(Range)	Mean(*SD*)/Median(Range)	*F/H*	GAD vs HC	MDD vs HC	GAD vs MDD
Age (years)	29.85(7.56)	27.97(8.16)	26.53(9.29)	*F_2,89_ =* 1.14	>0.13	>0.49	>0.40
Education (years)	14.87(3.30)	13.22(4.15)	14.09(3.20)	*F_2,89_ =* 1.34	>0.41	>0.34	>0.10
PSWQ	58.46(10.65) (N = 24)	62.34(8.53) (N = 29)	40.00(9.26) (N = 34)	*F_2,86_ =* 50.41***	<0.001***	<0.001***	=0.42
BDI-II^a^	24.00(2.00–42.00) (N = 24)	33.00(12.00–49.00) (N = 29)	3.50(0.00–21.00) (N = 34)	*H =* 57.22***	<0.001***	<0.001***	=0.13
CTQ-total^a^	50.50(34.00–79.00) (N = 22)	55.00(34.00–81.00) (N = 28)	40.00(31.00–78.00) (N = 33)	*H =* 17.02***	=0.048*	<0.001***	=0.56
CTQ-EA^a^	7.00(5.00–16.00)	8.00(5.00–22.00)	6.00(5.00–17.00)	*H =* 8.89*	>0.99	=0.012*	=0.16
CTQ-EN^a^	15.00(6.00–22.00)	18.00(6.00–25.00)	9.00(5.00–16.00)	*H =* 23.67***	=0.006***	<0.001***	=0.70
CTQ-PA^a^	5.00(5.00–15.00)	6.00(5.00–12.00)	5.00(5.00–12.00)	*H =* 0.79	>0.99	>0.99	>0.99
CTQ-PN^a^	15.50(10.00–21.00)	16.00(10.00–23.00)	14.00(10.00–22.00)	*H =* 4.36	=0.32	=0.18	>0.99
CTQ-SA^a^	6.00(5.00–13.00)	5.00(5.00–11.00)	5.00(5.00–11.00)	*H =* 3.24	=0.22	>0.99	=0.78

### 2.2 Experimental paradigm

Participants underwent a validated affective (linguistic) go/no-go fMRI paradigm that has been developed to explore the neurocircuitry underlying the interaction between emotional context and response inhibition (Goldstein et al., 2007, Protopopescu et al., 2005). The paradigm was designed as mixed event-related block design and behavioral responses were based on orthographical cues: participants were required to perform a button-press for words in normal font (go trial) and to inhibit this response to italicized font words (no-go trial). The emotional context of response inhibition was manipulated by employing words of different valence (negative, neutral, positive) per block (details see Supplementary Information).

### 2.3 MRI data acquisition and processing

MRI data was acquired on a 3 Tesla MRI system using standard acquisition parameters and preprocessed using SPM12 standard preprocessing routines (details Supplementary Information).

### 2.4 Statistical analysis and thresholding

Demographic data and anxiety symptom load between the groups were examined via one-way analysis of variance (ANOVA) and chi-square tests. Due to the non-normal distribution of BDI scores and CTQ scores (*p*s < 0.001, Shapiro-Wilk), Kruskal-Wallis tests were employed to examine group differences for these scales. Due to the non-normal distribution of the behavioral data (*p*s < 0.02, Shapiro-Wilk), nonparametric test were employed. To this end, nonparametric ANOVA-type analyses in nparLD with emotional valence × group as factors were conducted to analyze response times of correct Go trials. Accuracy rates were examined using ANOVA-type analyses in nparLD with trial category (go trials vs no-go trials) × emotional valence × group as factors. All post-hoc analyses employed appropriate Bonferroni adjustment.

For the analyses of the fMRI data the primary contrasts of interest [(a) for negative valence: [(neg vs. neu) × (no-go vs. go)], and (b) for positive valence: [(pos vs. neu) × (no-go vs. go)] were subjected to group-level random-effects analysis. Correspondingly, the statistical thresholds were set at *p*_FWE-cluster_ < 0.025 (0.05/2, corrected for the two valence contrasts). A whole-brain voxel-wise analysis examined differences between the diagnostic categories (MDD, GAD, and HC) using a one-way-ANOVA design (columns in the design matrix representing the GAD, MDD, and HC group) with gender and age as covariates in SPM12. Significant main effects of group were followed up by voxel-wise post-hoc independent *t*-tests that directly compared the three diagnostic groups. The voxel-wise statistics were performed on the whole brain level using a cluster-level Family-Wise Error (FWE) correction. To account for multiple analyses thresholding for the ANOVA models examining between group differences were adopted with respect to the two valence contrasts that were examine (*p*_FWE-cluster_ < 0.025; 0.05/2, corrected for two contrasts) and thresholding for the post-hoc independent *t*-tests was adopted to the number of groups (*p*_FWE-cluster_ < 0.017; 0.05/3, corrected for three groups). In line with recommendations for the application of cluster-level correction approaches an initial cluster forming threshold of *p* < 0.001 was employed (Slotnick, 2017, Woo et al., 2014).

In line with our previous studies (Xu et al., 2020b, Xu et al., 2020a) the categorical analysis was flanked by a subsequent follow-up dimensional analytic approach that examined associations between the observed categorical differences on the group level and MDD (BDI II scores) or GAD (PSWQ scores) symptom load, respectively, in the entire sample.

## 3 Results

### 3.1 Demographic data

After initial quality assessment of the data 26 patients with GAD, 30 patients with MDD and 34 HCs were included in the final analysis (detailed exclusion procedure see Supplementary Fig. S2). Participants in the GAD, MDD, and HC groups were of comparable age (*p* = 0.33), gender distribution (*p* = 0.06), and education level (*p* = 0.27). Some patients (one HC) reported being too exhausted to continue with the self-report questionnaire following the MRI assessments. The number of subjects for the GAD and MDD group therefore varies from 26 to 24 and 30 to 29 (BDI II, PSWQ), 26 to 22 and 30 to 28 (CTQ) respectively. Importantly, testing differences in the ratio of participants that discontinued the self-reported questionnaires did not reveal significant differences between the patient groups (Chi-square test, all *p*s > 0.05, detailed numbers provided in Table 1). Kruskal-Wallis analysis for depressive symptom load revealed a significant main effect of group (BDI-II, H = 57.22, *p* < 0.001), with post-hoc analyses indicating that depressive symptom load was higher in both GAD and MDD patients compared to HC, but not significantly different between the two patient groups (*p* value, GAD vs HC < 0.001, MDD vs HC < 0.001, GAD vs MDD = 0.13). Examining GAD symptom load revealed a significant main effect of group (PSWQ, *F*_2,86_ = 50.41, *p* < 0.001, *η^2^_p_ =* 0.55) with GAD symptom load being significantly higher in both patient groups relative to HC, but not significantly different between the two patient groups (*p* value, GAD vs HC < 0.001, MDD vs HC < 0.001, GAD vs MDD = 0.62, details see Table 1).

### 3.2 Behavioral results

**Fig. 1:**
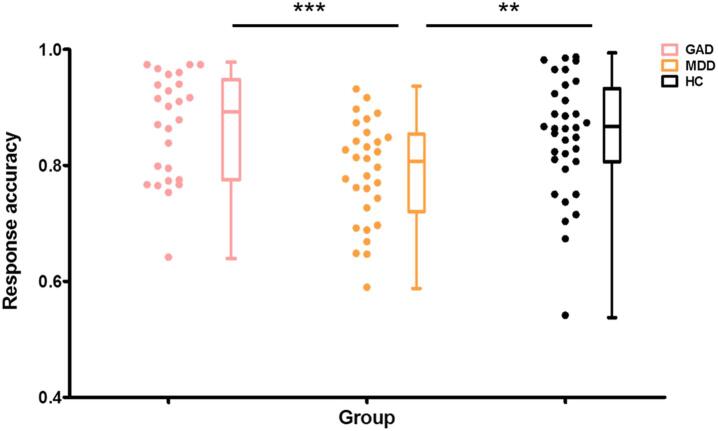
Response Accuracy in GAD, MDD and HC groups. **p < 0.01; ***p < 0.001, Bonferroni-corrected.

Examination of response accuracy revealed a significant main effect of group (*F*_2,83_ = 7.71, p < 0.001) with subsequent post-hoc tests with Kruskal-Wallis test demonstrating that across emotional contexts MDD patients made significantly less accurate responses compared to both, GAD patients (Z = 3.98, *p* < 0.001) and HC (Z = 3.21, *p* = 0.004), whereas GAD did not differ from HC (Z = 1.01, *p* = 0.94, Fig. 1). Moreover, a significant main effect of trial category suggested that all participants responded more accurately for go trials as compared to no-go trials (Z = 13.6, *p* < 0.001, Wilcoxon test, two-tailed). A significant main effect of emotional valance reflected that all participants made less accurate responses to positive as compared to negative trials (Z = 2.64; *p* < 0.05). No other main or interaction effects with respect to accuracy reached significance (*p*s > 0.17). No significant main or interaction effects were observed in the analysis of response times.

### 3.3 Neuroimaging results

**Fig. 2:**
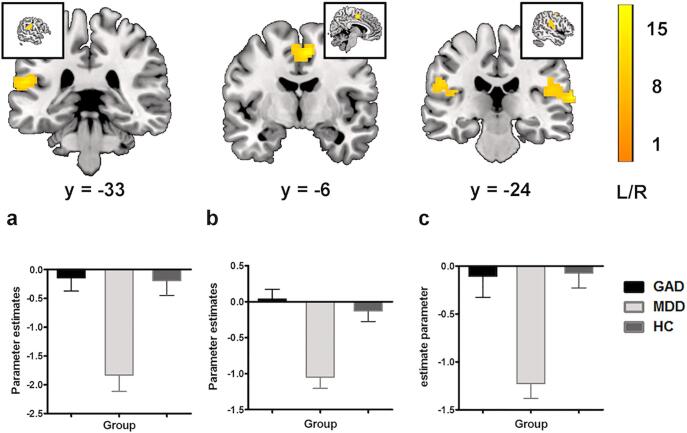
Main effect of diagnostic group (GAD, MDD and HC) for the interaction effect between negative emotional and inhibitory response [(neg – neu) × (no-go – go)]. All effects survived the family-wise error (FWE) correction for multiple comparisons (*p*_FWE_ < 0.025 with an initial cluster forming threshold of *p* < 0.001). The color bar codes the *F* value. For visualization purpose the extracted estimates for the interaction effect between negative emotional and inhibitory response [(neg – neu) × (no-go – go)] are displayed for each group, left postcentral gyrus/ supramarginal gyrus (2a), bilateral middle cingulate (2b), and right postcentral gyrus/precentral gyrus/supramarginal gyrus (2c). L/R, left/right.

**Fig. 3:**
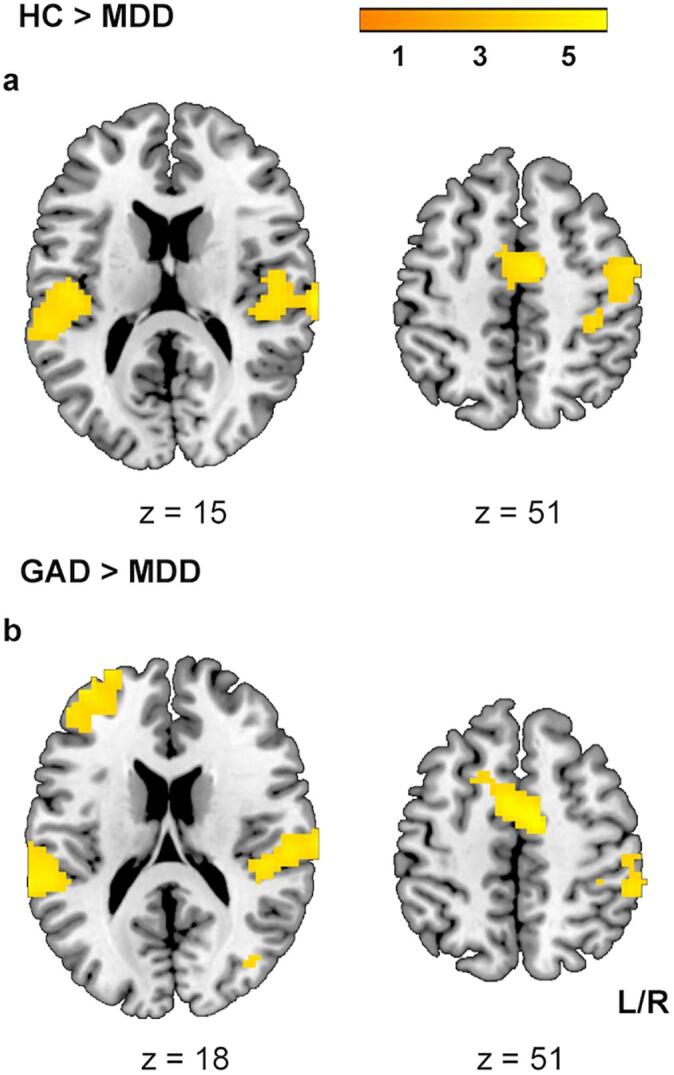
Comparisons between patients with MDD and patients with GAD and HC for the interaction effect between negative emotional and inhibitory response [(neg – neu) × (no-go – go)]. All effects survived the family-wise error (FWE) correction for multiple comparisons (*p*_FWE_ < 0.017 with an initial cluster forming threshold of *p* < 0.001). The color bar codes the *t* value. L/R, left/right.

Examining the positive emotional context revealed no significant differences between the groups. In contrast, the voxel-wise whole-brain ANOVA examining the negative context revealed a significant interaction effect involving the factor group in a widespread bilateral sensory-motor and cognitive control network, encompassing the left postcentral gyrus/supramarginal gyrus (MNI [-57–33 18], *p*_FWE-cluster_ = 0.007, k = 164, *F*_2,85_ = 13.14), right postcentral gyrus/precentral gyrus/supramarginal gyrus (MNI [69–24 12], *p*_FWE-cluster_ < 0.001, k = 248, *F*_2,85_ = 15.03), and the bilateral middle cingulate (MNI [6–6 51], *p*_FWE-cluster_ = 0.005, k = 176, *F*_2,85_ = 14.23; Fig. 2). The main effect of group remained robust after including BDI scores, gender, and age as covariates (*p*s_FWE-cluster_ < 0.025, details see Supplementary Fig. S3). A subsequent direct comparison of the three groups by means of voxel-wise SPM12 independent t-tests revealed significantly attenuated engagement of these regions in MDD patients compared to both, HC and GAD (details see Fig. 3a, Fig. 3b), whereas GAD patients exhibited no differences compared to HC, indicating that the interaction effect was driven by altered neural activation in the MDD patients. Moreover, the direct comparison between GAD and MDD groups additionally revealed significantly increased recruitment of the left dorsal lateral prefrontal cortex (DLPFC, MNI [−39 54 21], *p*_FWE-cluster_ = 0.004, k = 251, *t*_85_ = 4.34) in the GAD relative to MDD patients Fig. 3b).

To further disentangle the complex interaction effect and to determine alterations during inhibitory control in negative contexts an additional voxel-wise ANOVA focused on the contrast [neg no-go vs. neu no-go]. The one-way-ANOVA model in SPM12 (columns in the design matrix representing the GAD, MDD, and HC group) with gender and age as covariates revealed a significant main effect of group during inhibitory control in negative contexts encompassing the network described above (*p*_FWE-cluster_ < 0.025, details see Supplementary Fig. S4a). A subsequent direct comparison of the three groups by means of voxel-wise SPM12 independent t-tests revealed significantly attenuated engagement of these regions in MDD patients compared to both, HC and GAD (displayed in Supplementary Fig. S4b, S4c), whereas GAD patients exhibited no differences compared to HC, further emphasizing inhibitory control-specific neurofunctional alterations in MDD. In addition, we conducted an identical follow-up analysis for the control condition in the negative context [neg go vs. neu go], and the one-way-ANOVA model in SPM12 revealed a significant main effect of group in the right supramarginal gyrus (MNI [54–39 48], *p*_FWE-cluster_ = 0.01, k = 157, *F*_2,85_ = 12.24, details see Supplementary Fig. S5a). Post hoc direct comparisons between the three groups by means of voxel-wise SPM12 independent t-tests revealed significantly increased engagement of this region in MDD patients compared to GAD patients, while none of the patients groups differed from the HC (displayed in Supplementary Fig. S5b)

### 3.4 Dimensional analysis

In line with our previous studies (Xu et al., 2020b) the identified categorical between-group differences were followed up by a dimensional analysis approach. To this end, associations between BDI II scores and the identified behavioral (accuracy), and neural alterations (extracted parameter estimates) in the entire sample, were examined by linear models (FSL PALM-alpha toolbox (https://fsl.fmrib.ox.ac.uk/fsl/fslwiki/PALM, Permutation Analysis of Linear Models, number of permutations = 10,000) including GAD symptom load as covariate. No significant associations were observed (all *p*s > 0.05).

## 4 Discussion

The present study aimed at determining disorder-specific behavioral and neurofunctional dysregulations in emotional context-specific inhibitory control in MDD and GAD patients. To this end we employed a validated affective go/no-go fMRI paradigm in unmedicated MDD patients, GAD patients and HC. On both, the behavioral and neural level we found supporting evidence for disorder-specific impairments, such that MDD patients exhibited generally impaired inhibitory control in terms of reduced accuracy rates as compared to both HC and GAD patients, while GAD patients did not differ from HC. On the neural level specifically MDD patients demonstrated attenuated recruitment of a broad bilateral network encompassing inferior/medial parietal and posterior frontal as well as mid-cingulate regions during inhibitory control in the negative context, suggesting disorder- and emotional context-specific neurofunctional deficits. Further examination of disorder-specific alterations revealed that GAD patients exhibited a stronger engagement of the left DLPFC relative to MDD patients.

On the behavioral level, the observed pattern in the present study partly resembles findings in previous studies (Erickson et al., 2005, Harfmann et al., 2019, Yu et al., 2015) such that MDD patients showed lower accuracy while GAD patients exhibited comparable accuracy with HC. However, while the previous studies reported emotion-specific inhibitory control deficits in MDD patients the present study found a general impairment in no-go accuracy irrespective of emotional context. The differences between the studies may be explained in the sample characteristics, such that previous studies emphasized a focus on emphasized a ecological and clinical validity by including MDD patients with a history of previous episodes and current pharmacological treatment (Erickson et al., 2005, Harfmann et al., 2019). In contrast, the present study aimed at specifically determining disorder-specific neurobiological mechanisms while controlling for these factors. Together, the findings may indicate comparably subtle and rather general cognitive impairments during early and unmedicated stages of MDD.

MDD patients specifically exhibited decreased recruitment of the parietal and posterior frontal regions during trials that required inhibitory control of the prepotent motor response, indicating specific neurofunctional deficits during cognitive control. Together with the prefrontal systems, the parietal and posterior frontal regions constitute the fronto-parietal network which has been consistently involved in cognitive control processes, including inhibition of prepotent motor responses during go/no-go paradigms (Goghari and MacDonald, 2009, Niendam et al., 2012). Within this network the precentral / postcentral gyrus has been specifically associated with mild emotional interference during cognitive control (Song et al., 2017) and the parietal cortex is involved in biases relevant to stimulus–response associations, while prefrontal regions constitute a more domain-general network regulating emotional and cognitive interference (Chen et al., 2018, Xu et al., 2016). On the one hand the present findings of deficient neural engagement during cognitive control in negative contexts generally align with previous studies reporting context-specific neural alterations in MDD patients as compared to controls (Colich et al., 2016). On the other hand, these previous studies reported alterations in prefrontal regions, specifically dorsolateral and ventrolateral regions, whereas the present study found alterations in parietal, posterior frontal, and cortical midline regions, specifically the supramarginal, postcentral and precentral gyrus, as well as the MCC. The diverging results with respect to the specific location of the emotional context-specific neurofunctional alterations in MDD may be partly explained by the differences in the sample characteristics. Thus, previous studies were conducted in MDD patients with recurrent episodes of depression and under anti-depressive medication while the present study examined unmedicated first episode patients. In addition to progressive emotional and cognitive dysregulations during the course of the disorder, progressive changes in structural integrity, particularly in prefrontal regions, have been reported in MDD (Serra-Blasco et al., 2013). Together with the present results this may suggest that during the progressive course of the disorder or pharmacological treatment neural alterations shift from posterior to more prefrontal regions.

MDD patients in the present study additionally exhibited deficient recruitment of the bilateral MCC during inhibitory control in the negative context. A recent transdiagnostic neuroimaging meta-analysis reported altered activity in this region as well as core regions of the fronto-parietal cognitive control network across different cognitive control paradigms and psychiatric disorders (McTeague et al., 2017). A recent study reported that patients with treatment resistant depression who underwent focal bilateral anterior cingulotomy targeting the MCC subsequently exhibit specific impairments in recognizing negative stimuli and in inhibitory control of prepotent stimulus–response contingencies while exhibiting enhanced interference sensitivity (Tolomeo et al., 2016). This finding suggests a critical role of the MCC for engaging cognitive control processes in the presence of negative stimuli to optimize goal directed behavior. Furthermore, recent overarching reviews suggest that – together with the anterior portion of the cingulate – the MCC constitutes a highly integrative hub bridging negative emotion processing, pain, and cognitive control with motor systems executing goal-directed behavior (Kragel et al., 2018, Shackman et al., 2011, Tolomeo et al., 2016). Together, these findings suggest a deficient recruitment of a network engaged in the integration of emotional inference and motor systems during inhibitory control in MDD.

In contrast to previous studies that reported dimensional associations between depressive symptom-load and altered intrinsic brain architecture (Oathes et al., 2015, Xu et al., 2020a) as well as altered pain empathic insula reactivity (Xu et al., 2020b) across GAD and MDD patients the present study did not reveal significant associations with respect to the behavioral and neurofunctional alterations observed in the categorical analysis. Together with a visual inspection of the extracted parameter estimates from the categorical approach (see Fig. 2, lower panel), this suggests rather categorical differences between MDD and GAD in the domain of inhibitory deficits in negative contexts which may indicate a particular diagnostic specificity of dysfunctions in this domain.

A direct comparison of the patient groups with respect to neural activation during cognitive control in negative contexts further revealed higher DLPFC activation in GAD relative to MDD patients. The DLPFC represents a core region of the domain general cognitive control network and subserves inhibitory control, cognitive flexibility, and working memory (Niendam et al., 2012). Previous studies that targeted the left DLPFC with non-invasive brain stimulation techniques reported improved cognitive control in healthy individuals (Nejati et al., 2018) and improved cognitive control in emotional contexts in MDD patients (Bermpohl et al., 2006, Boggio et al., 2007), suggesting a contribution of this region to cognitive control performance.

Findings of the present study need to be considered in the context of limitations. Firstly, in the present study unspecific behavioral deficits and emotion-specific neurofunctional alterations were observed in MDD. The diverging behavioral and neural results may reflect different analytic approaches, such that the fMRI analyses focused on the contrasts between each emotion with neutral to control for confounding effects of unspecific decreases in attention or processing speed in MDD on neural activity (Rock et al., 2014, Snyder, 2013). Secondly, to control for important confounders such as treatment or progressive dysregulations during the course of the disorder, we employed strict enrollment criteria, which came at the cost of only a minority of patients in two large psychiatric hospitals being eligible for enrollment thus leading to a moderate sample size. Thirdly, although it is suggested that there are significant differences in depression and anxiety between males and females (DeVido et al., 2009, Fadok et al., 2018), the relatively small sample size did not allow us to further explore gender differences. Therefore, potential gender differences need to be explored in future studies. Fourthly, although the primary diagnosis of GAD and MDD was determined by experienced clinical psychiatrists some patients (MDD n = 7 in the GAD group, GAD n = 6 in the MDD group) exhibited a secondary GAD or MDD co-morbidity according to the M.I.N.I. interview. Finally, the use of a blocked design in the current study does not allow to further disentangle the contributing factors within a block.

## 5 Conclusion

Overall, the findings from the present study suggest disorder-specific neurofunctional alterations during inhibitory control in negative emotional contexts in MDD, specifically a deficient engagement of a broad bilateral network encompassing inferior/medial parietal and posterior frontal as well as mid-cingulate regions. Although GAD patients did not demonstrate deficits on the behavioral and neural level in comparison to healthy controls, stronger recruitment of the DLPFC as compared to MDD patients may point to a compensatory mechanism on the neural level that facilitates intact inhibitory control in GAD.

## Authors contribution

Conceptualization was provided by Benjamin Becker and Keith Kendrick. Administration, investigation, and methodology was provided by Congcong Liu, Xiaolei Xu and Lizhu Luo. Funding was procured by Benjamin Becker and Jing Dai. Investigation was conducted Congcong Liu, Xiaolei Xu, Yuanshu Chen and Fei Xin. Resources are provided by Bo Zhou, Jing Dai, Zhili Zou, Yulan Huang and Jinyu Wang. Validation, software visualization and formal analysis were completed by Benjamin Becker, Congcong Liu, Ziyu Qi, Qian Zhuang, Xinqi Zhou, Feng Zhou and Huafu Chen. Finally, Congcong Liu and Benjamin Becker wrote the manuscript. Benjamin Becker and Keith Kendrick reviewed the manuscript, and also provided edits.

## Declaration of Competing Interest

The authors declare that they have no known competing financial interests or personal relationships that could have appeared to influence the work reported in this paper.
