# Supplementary material for: Disorder- and emotional context-specific neurofunctional alterations during inhibitory control in generalized anxiety and major depressive disorder
Source: Neuroimage Clin. 2021 Apr 3;30:102661. doi: 10.1016/j.nicl.2021.102661 (PMC8060548; doi:10.1016/j.nicl.2021.102661)

**Supplementary Information**

**Liu et al.,**

**Supplementary methods**

**Participants**

Patients were recruited at the Sichuan Provincial People’s Hospital and The Fourth People’s Hospital of Chengdu (Chengdu, China). To facilitate diagnostic accuracy a two-step approach was employed: (1) Diagnoses according to DSM-IV (Sichuan Provincial People’s Hospital) or ICD-10 (Fourth People’s Hospital of Chengdu) criteria were initially determined through clinical interviews by experienced psychiatrists, and next (2) independently confirmed by an experienced clinical psychologist by means of the Mini International Neuropsychiatric Interview (M.I.N.I.) for DSM-IV. Exclusion criteria for participants were: (1) current/history of the following DSM axis I disorders: post-traumatic stress, feeding and eating, substance use, bipolar disorder and mania, (2) current/history of medical or neurological disorders, (3) acute (within six weeks before enrollment) or chronic medication use, (4) suicidal ideation, (5) MRI contraindication. All patients in the GAD group received the primary diagnosis of GAD whereas all patients in the MDD group received the primary diagnosis of MDD across the diagnostic approaches. In the M.I.N.I interview n = 7 of the 35 patients in the GAD group additionally fulfilled the criteria for a co-morbid MDD, while n = 6 of 37 patients in the MDD group additionally fulfilled the criteria for a co-morbid GAD. To facilitate data quality, and reduce the burden for the participants, participants were explicitly asked whether their current status (e.g. exhaustion, emotional state) allowed proceeding with the assessments before each assessment (e.g. MRI, questionnaires). The study was part of a larger project on common and disorders-specific alterations in MDD and GAD, the present paradigm was preceded by resting state fMRI acquisition (X. Xu, Dai, Chen, et al., 2020a) and followed by a pain empathy paradigm (X. Xu, Dai, Liu, et al., 2020b).

Experimental paradigm

A total of 54 words written in Chinese were used (18 per emotional context condition). Words were matched across the valence conditions for word frequency and length (length of each word = 4 characters). A pre-study in an independent sample of n = 18 subjects demonstrated a high emotional category-specificity of the words as well as comparable imaginability, intensity (positive, negative) and word frequency between the conditions (details see **Supplementary Table S1**).

The stimuli were presented over two runs and each run comprised two blocks of the six permutations of task (go vs. no-go) and emotional context (positive, neutral, negative): neutral go (neu go), neutral no-go (neu no-go), negative go (neg go), negative no-go (neg no-go), positive go (pos go), positive no-go (pos no-go). Order of presentation was counterbalanced. Go blocks included 18 words in normal font (100% Go trials) and no-go blocks included 12 normal font words (66.7% go trials) and 6 italicized font words (33.3% no-go trials), presented in a pseudorandomized order. All blocks were preceded by a brief instruction (‘For normal font please respond by button press, otherwise, no response’) that was presented for 4s (schematic depiction see **Supplementary figure S1**). Each word was presented for 300ms followed by a 900ms interstimulus interval (total block duration=21.6 sec). Each block was followed by a low-level inter-block interval of 16s. Total task duration was 17min. To ensure that all participants understood the task paradigm they underwent a brief practice run with different words before the experiment.

MRI data acquisition

MRI data were acquired on a 3 Tesla GE MR750 system (General Electric Medical System, Milwaukee, WI, USA). To exclude subjects with apparent brain pathologies and improve normalization of the functional time series, T1-weighted high-resolution anatomical images were acquired with a spoiled gradient echo pulse sequence, repetition time (TR) = 6 ms, echo time (TE) = minimum, flip angle = 9°, field of view (FOV) = 256 × 256 mm, acquisition matrix = 256 × 256, thickness = 1 mm, number of slice = 156. For the functional MRI timeseries a total of 512 functional volumes were acquired using a T2*-weighted Echo Planar Imaging (EPI) sequence (TR = 2000 ms, TE = 30 ms, FOV = 240 × 240 mm, flip angle = 90°, image matrix = 64 × 64, thickness/gap = 3.4/0.6mm, 39 axial slices with an interleaved ascending order).

MRI data processing

MRI data was processed and analyzed using Statistical Parametric Mapping (SPM12; Wellcome Department of Cognitive Neurology, Institute of Neurology, London, United Kingdom). For each subject and run, the first 6 functional volumes were discarded to allow magnet-steady images. The remaining functional images were preprocessed using standard preprocessing procedures including: slice timing, realigning to correct for head motion, a two-step normalization to Montreal Neurological Institute (MNI) standard space including co-registration to the T1-weighted structural images and application of the segmentation parameters obtained from segmenting the structural images to the functional time-series (interpolated at 3×3×3mm voxel size) and spatial smoothing using an 8mm full-width at half-maximum (FWHM) Gaussian kernel. For statistical analysis a two-step general linear model (GLM) approach was employed. At the single subject level an event-related general linear model (GLM) was employed including condition-specific regressors modelling the six experimental conditions and the six head motion parameters. Regressors for the experimental conditions were convolved with the default SPM hemodynamic response function (HRF). The design matrices additionally included a high pass filter to control for low frequency components and a first-order autoregressive model (AR[1]) to account for autocorrelation in the time-series.

**Reference**

Xu, X., Dai, J., Chen, Y., Liu, C., Xin, F., Zhou, X., Zhou, F., Stamatakis, E.A., Yao, S., Luo, L., Huang, Y., Wang, J., Zou, Z., Vatansever, D., Kendrick, K.M., Zhou, B., Becker, B., 2020a. Intrinsic connectivity of the prefrontal cortex and striato-limbic system respectively differentiate major depressive from generalized anxiety disorder. Neuropsychopharmacol. Off. Publ. Am. Coll. Neuropsychopharmacol. https://doi.org/10.1038/s41386-020-00868-5

Xu, X., Dai, J., Liu, C., Chen, Y., Xin, F., Zhou, F., Zhou, X., Huang, Y., Wang, J., Zou, Z., Li, J., Ebstein, R.P., Kendrick, K.M., Zhou, B., Becker, B., 2020b. Common and Disorder-Specific Neurofunctional Markers of Dysregulated Empathic Reactivity in Major Depression and Generalized Anxiety Disorder. Psychother. Psychosom. 89, 114–116. https://doi.org/10.1159/000504180

**Table S1. Results from emotional valence** (yes and no rating of the fit to the corresponding category), intensity (1-9 scale), and imaginability (1-9 scale) ratings of the word stimuli in an independent sample (n = 18) as well as word frequency.

|  | **Stimuli** | | | ***F p*** | |
| --- | --- | --- | --- | --- | --- |
| **Measurements** | **Positive** | **Negative** | **Neutral** |  |  |
| **Emotional valence rating** | 94.58% ± 1.07% | 96.00% ± 1.07% | 96.21% ± 0.73% | 0.95 | 0.35 |
| **Frequency** | 1965.58 ± 340.04 | 1007.24 ± 446.22 | 1725.54 ± 288.05 | 1.87 | 0.16 |
| **Intensity** | 5.73 ± 0.17 | 6.15 ± 0.23 |  | 1.47 (*t*) | 0.15 |
| **Imagination** | 5.76 ± 0.23 | 6.07 ± 0.22 | 5.70 ± 0.12 | 1.02 | 0.37 |

**Figure S1.** Schematic display and timing parameters of the emotional linguistic go/nogo paradigm. A negative go block and positive no-go block are depicted for the emotional linguistic go/no-go task.


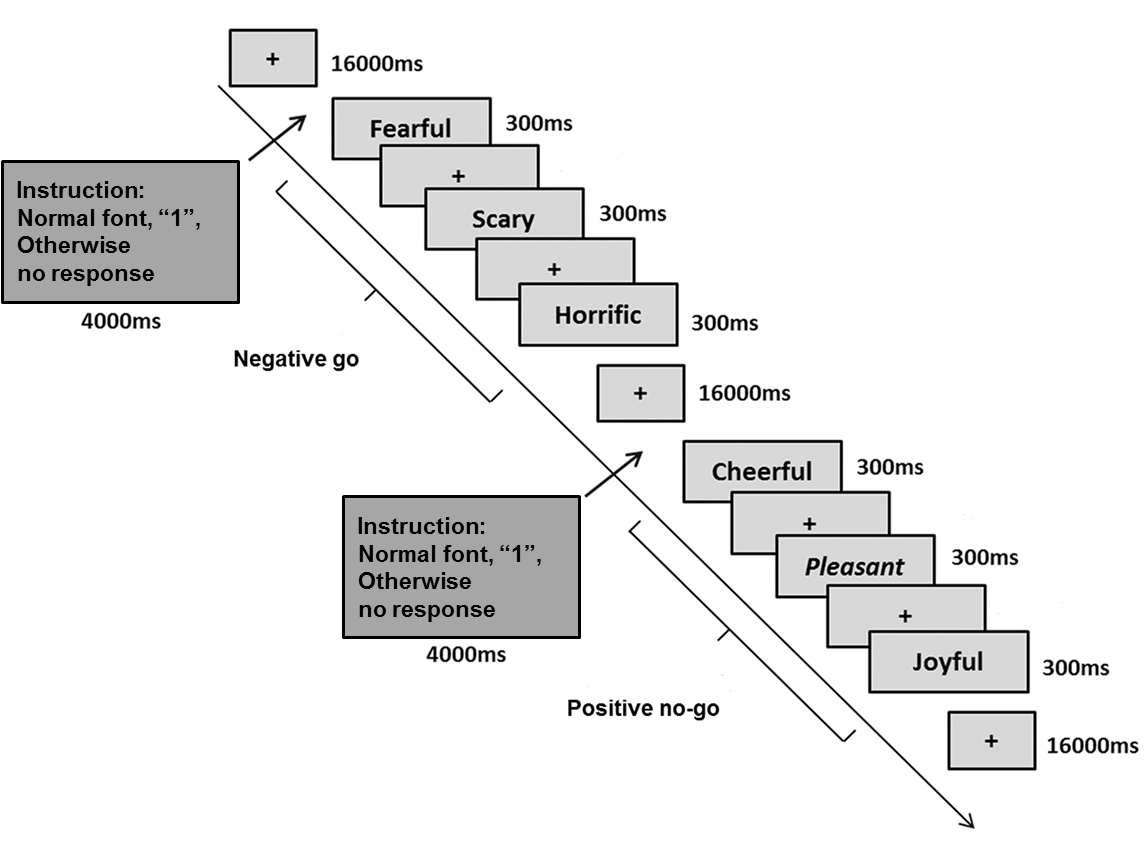


**Figure S2** **Flow diagram displaying exclusion of participant and rationale for exclusion**


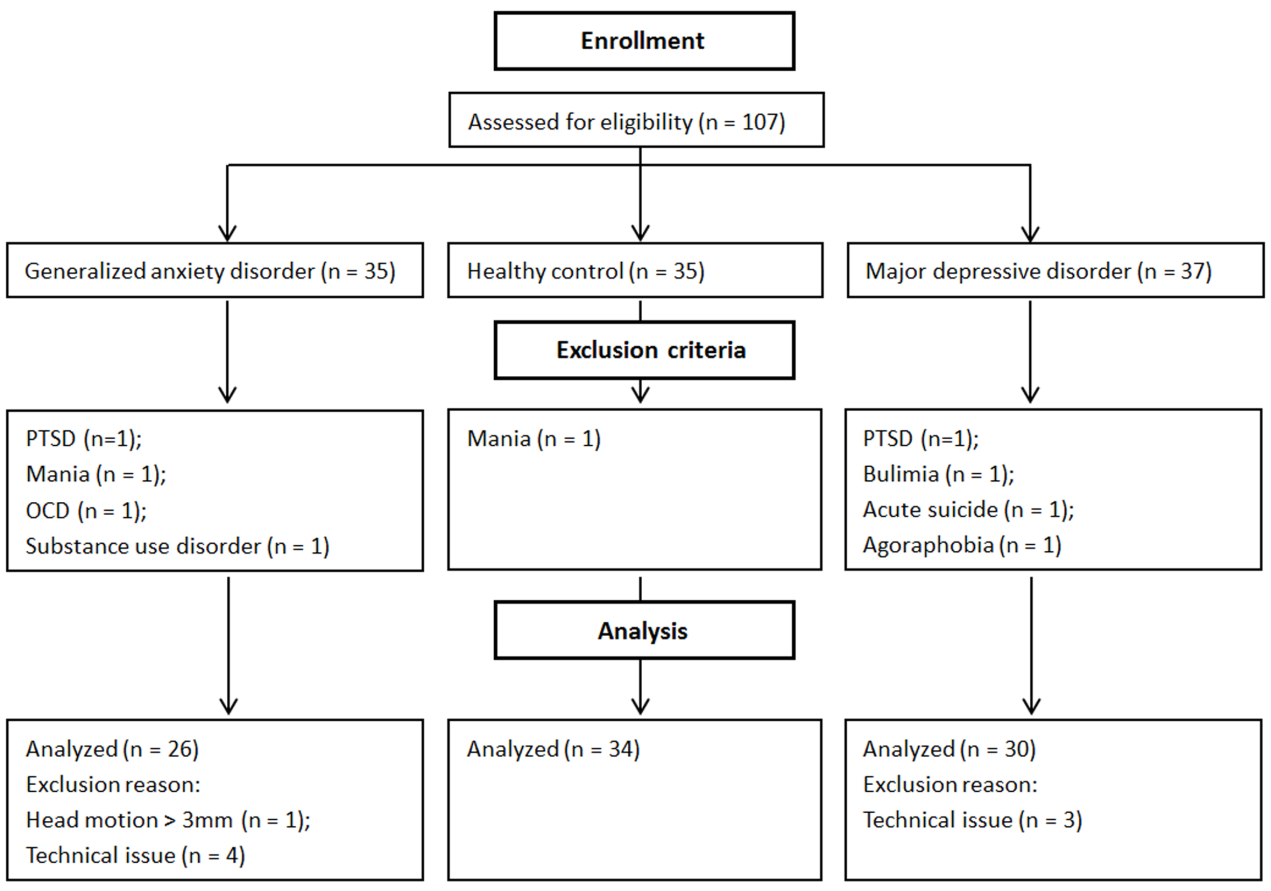


**Figure S3** Main effect of diagnostic group (GAD, MDD and HC) after including BDI scores as a covariate for the interaction effect between negative emotion context and inhibitory control [(neg – neu) × (no-go – go)]. Results are displayed at family-wise error (FWE) correction for multiple comparisons (*p*_FWE_ < 0.025 with an initial cluster forming threshold of *p* < 0.001). L, left; R, right.

**
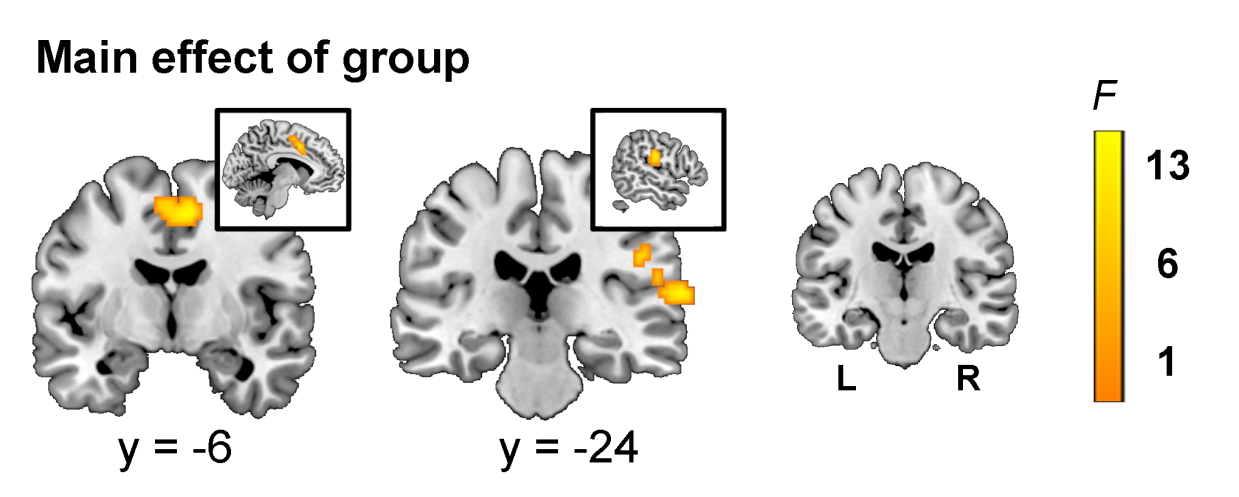
**

**Figure S4.** Results from the ANOVA examining group differences during inhibitory control in the negative context. Displayed is the main effect of diagnostic group (GAD, MDD and HC) (a) and post hoc results from the between-group comparison comparing patients with major depression (MDD) with healthy controls (HC) (b) or patients with generalized anxiety disorder (GAD), respectively (c). Results are displayed at family-wise error (FWE) correction for multiple comparisons (*p*_FWE_ < 0.025 with an initial cluster forming threshold of *p* < 0.001). L, left; R, right.


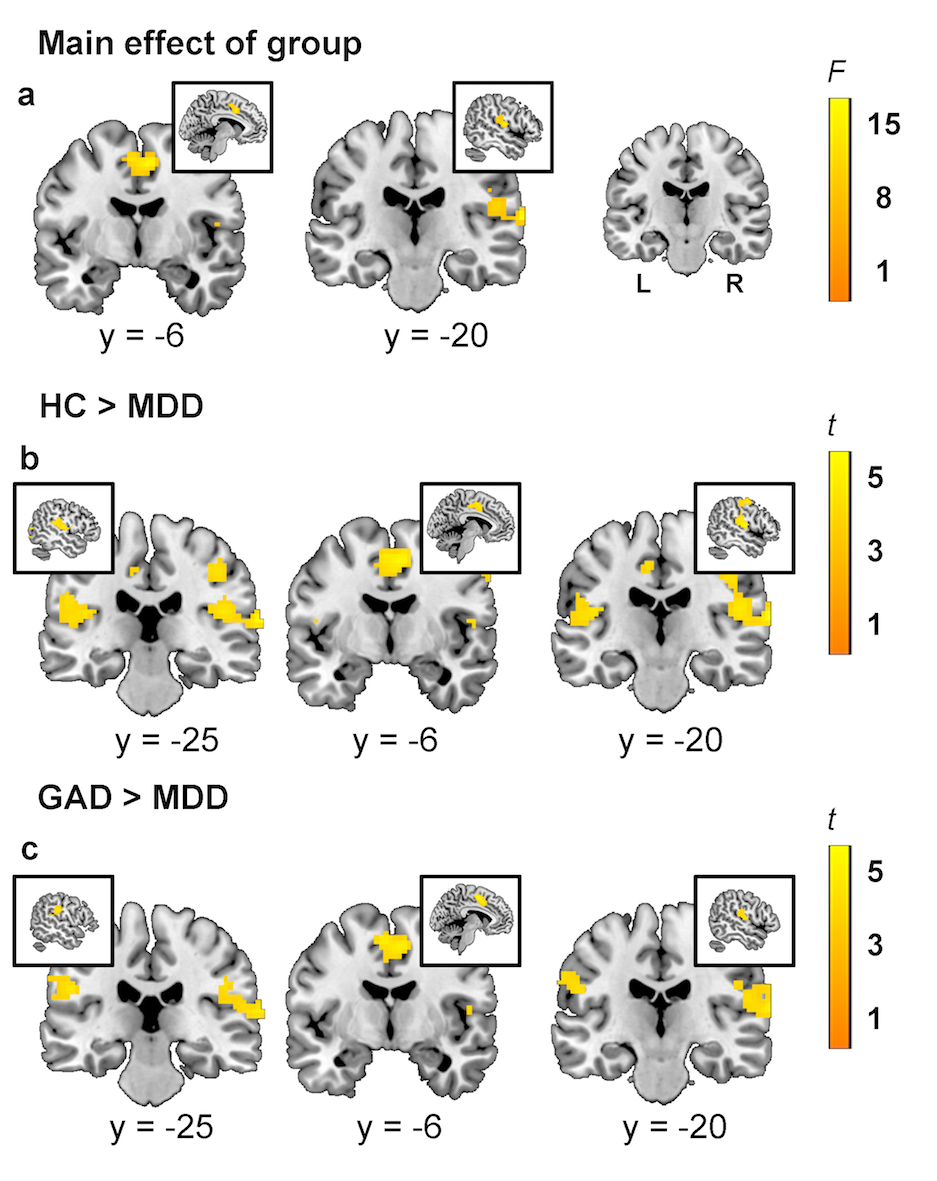


**Figure S5.** Results from the ANOVA examining group differences during the control condition in negative context. Displayed is the main effect of diagnostic group (GAD, MDD and HC) (a) and post hoc results from the between-group comparison comparing patients with major depression (MDD) with patients with generalized anxiety disorder (GAD) (b). Results are displayed at family-wise error (FWE) correction for multiple comparisons (*p*_FWE_ < 0.017 with an initial cluster forming threshold of *p* < 0.001). L, left; R, right.


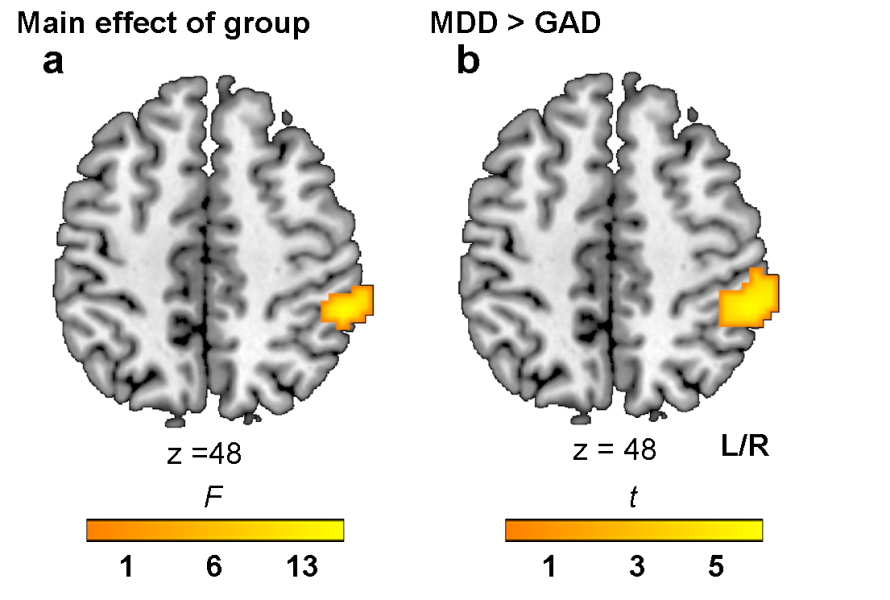

Supplement: Supplementary data 1 [file mmc1.docx]
